# *Hoya pyrifolia* (Apocynaceae), a new species from south-western Yunnan, China

**DOI:** 10.3897/phytokeys.174.60137

**Published:** 2021-02-02

**Authors:** Er-Feng Huang, Gang Yao, Ri-Hong Jiang, Lei-Lei Yang, Wang Xi, Zhong-Shuai Zhang, Xian-Chun Zhang

**Affiliations:** 1 Guangxi Nanning Roy Garden Co. Ltd., Nanning 530227, Guangxi, China Guangxi Nanning Roy Garden Co. Ltd. Nanning China; 2 College of Forestry and Landscape Architecture, South China Agricultural University, Guangzhou 510642, China South China Agricultural University Guangzhou China; 3 State Key Laboratory of Systematic and Evolutionary Botany, Institute of Botany, Chinese Academy of Sciences, Beijing 100093, China Institute of Botany, Chinese Academy of Sciences Beijing China; 4 University of Chinese Academy of Sciences, Beijing 10049, China University of Chinese Academy of Sciences Beijing China; 5 Shenzhen Key Laboratory of Southern Subtropical Plant Diversity, Fairy Lake Botanical Garden, Shenzhen & Chinese Academy of Sciences, Shenzhen 518004, Guangdong, China Fairy Lake Botanical Garden, Shenzhen & Chinese Academy of Sciences Shenzhen China; 6 Kunming Institute of Botany, Chinese Academy of Sciences, Kunming 650201, Yunnan, China Kunming Institute of Botany, Chinese Academy of Sciences Kunming China; 7 State Key Laboratory of Tree Genetics and Breeding, Chinese Academy of Forestry, Beijing 100091, China Chinese Academy of Forestry Beijing China

**Keywords:** Apocynaceae, Asclepiodoideae, China, *
Hoya
*, taxonomy

## Abstract

*Hoya pyrifolia*, a new species of Apocynaceae from Yunnan Province, China, is described and illustrated. Results from phylogenetic analyses, based on combined DNA fragments of the nuclear ribosomal external transcribed spacer (ETS), intergeneric transcribed spacer (ITS) and three plastid DNA fragments (*matK*, *psbA-trnH* and *trnT-trnL*), showed that the new species was nested within a clade, including *Hoya* species distributed in the subtropical foothills of the Himalayas and the Tibet-Sichuan Plateau. Morphologically, the new species can be distinguished from its close relatives by its pyriform and slightly pubescent leaves, as well as the 4-flowered inflorescences.

## Introduction

*Hoya* R.Br., the wax plants, is a large genus circumscribed within the tribe Marsdenieae, subfamily Asclepiodoideae of Apocynaceae (Wanntorp et al. 2014). It includes over 300 species mainly distributed in the tropical and subtropical regions of Asia, Oceania and the Pacific Islands (Wanntorp et al. 2014), with ca. 40 species recorded in China (Li et al. 1995; Huang et al. 2020a). Several infrageneric classification systems of the genus have been proposed by different authors (Hooker 1885; Schlechter 1916; Burton 1995), but none of them was supported in phylogenetic analyses (Wanntorp et al. 2006; Wanntorp et al. 2014; Huang et al. 2020a).

During a field investigation in Yingjiang Hsien, Yunnan Province, China, in the summer of 2018, one of the authors (E.F. Huang) discovered one population of a *Hoya* species, which obviously is different from congeneric taxa recorded in China and adjacent countries. Later, a specimen representing the same species was collected again from another locality (viz. Longling Hsien) in south-western Yunnan. Detailed morphological comparison and specimen examination for all the *Hoya* species recorded in China and adjacent regions showed that the species is new to science, thus it is formally described and illustrated here as a new species. The phylogenetic position of the new species is studied based on analyses of a combined matrix including five DNA fragments from both plastid and nuclear genomes.

## Materials and methods

### Morphological study

Specimens of *Hoya* deposited in the herbaria CDBI, GH, HNWP, IBSC, KUN, P and PE were studied carefully in the present study. Field investigations of Chinese *Hoya* species were also conducted in recent years. Morphological characters of leaves, inflorescences and flowers of relevant species were photographed and measured. Herbarium abbreviations cited in the present study follow the Index Herbariorum (Thiers 2013 onwards).

### Phylogenetic study

To study the phylogenetic position of the new species within the genus *Hoya*, a phylogenetic study of the genus was performed, based on combined DNA fragments of the nuclear ribosomal external transcribed spacer (ETS), intergeneric transcribed spacer (ITS) and three plastid DNA regions (*matK*, *psbA-trnH* and *trnT-trnL*), following Huang et al. (2020a). Total genomic DNA of the new species was extracted from silica gel-dried leaves (voucher specimen: *E.F. Huang 1905009*, PE) using a Plant Genomic DNA Kit (Biomed, Shenzhen, China). Detailed information of primers of relevant DNA fragments used in Polymerase Chain Reaction (PCR) amplification and sequencing, as well as the procedures of PCR, can be found in Huang et al. (2020a). Based on morphological traits, the new species studied here seems to belong to clade I in Wanntorp et al. (2014). Thus, other species belonging to this clade sampled in previous studies (Wanntorp et al. 2014; Huang et al. 2020a) were included in our study, as well as representatives of other major clades of the genus. A species from the genus *Marsdenia* R.Br. was selected as outgroup, based on the phylogenetic framework reported in previous studies (Wanntorp et al. 2014). Detailed information of all species sampled and sequences used are available in Appendix 1.

Sequences were aligned using MAFFT 7.221 (Katoh and Standley 2013) and then three major datasets were constructed: the cpDNA dataset (including *matK*, *psbA-trnH* and *trnT-trnL*), the nrDNA dataset (including ETS and ITS) and the combined dataset including the five DNA fragments (ETS, ITS, *matK*, *psbA-trnH* and *trnT-trnL*). The three datasets were analysed with Bayesian Inference (BI) and Maximum Likelihood (ML). Detailed information about the parameter setting in BI and ML analyses is given in Huang et al. (2020b). The models of nucleotide substitution of each fragment used here were selected under the Akaike Information Criterion (AIC) using jModelTest v. 3.7 (Posada 2008): GTR+Γ for ETS, TIM1+Γ for ITS, TPM1uf+I+Γ for *matK*, TrN+Γ for *psbA-trnH* and TPM1uf+Γ for *trnT-trnL*.

## Results and discussion

**Figure 1.**
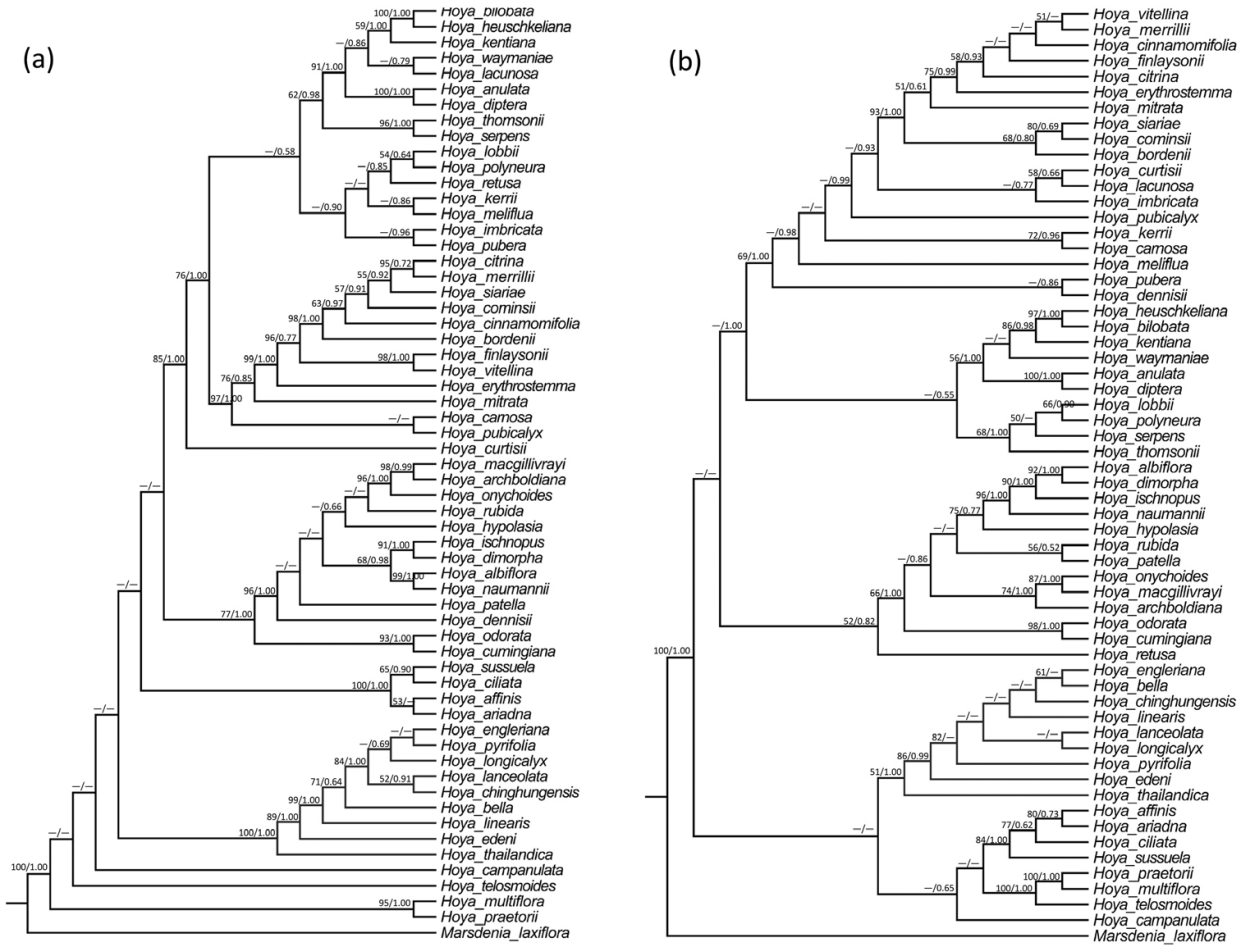
Maximum Likelihood (ML) tree of *Hoya* species inferred from the nrDNA (**a** including ETS and ITS) and cpDNA (**b** including *matK*, *psbA-trnH* and *trnT-trnL*) datasets. Bootstrap (BS) values ≥ 50% in ML analysis and posterior probability (PP) ≥ 0.50 in Bayesian Inference (BI) are indicated on the left and right of slanting bars above a phylogenetic node, respectively. Dashes denote that the phylogenetic node was not supported, the BS value is < 50% in the ML analysis or PP < 0.50 in the BI analysis.

**Figure 2.**
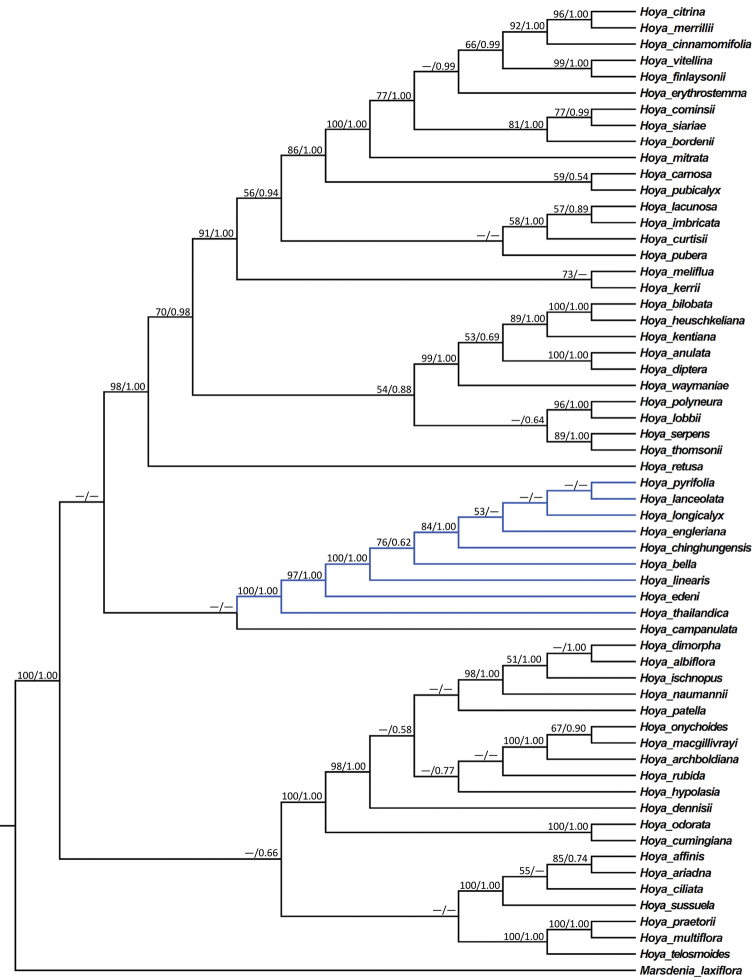
Maximum Likelihood (ML) tree of *Hoya* species inferred from the combined dataset of five DNA regions (ETS, ITS, *matK*, *psbA-trnH* and *trnT-trnL*). Bootstrap (BS) values ≥ 50% in ML analysis and posterior probability (PP) ≥ 0.50 in Bayesian Inference (BI) are indicated on the left and right of slanting bars above a phylogenetic node, respectively. Dashes denote that the phylogenetic node was not supported, the BS value is < 50% in the ML analysis or PP < 0.50 in the BI analysis.

The cpDNA dataset, the nrDNA dataset and the combined dataset contained 2482, 1393 and 3875 characters, respectively. Some major clades within the genus *Hoya* were recovered in the BI and ML analyses of the three datasets (Figures 1–2), but phylogenetic relationships amongst these major clades were inconsistent. However, conflicting phylogenetic nodes were all poorly supported [bootstrap support (BS) in ML analysis < 50% and/or posterior probabilities (PP) in BI analysis < 0.50] (Figures 1–2).

Results from both BI and ML analyses of the three major datasets all showed that the new species studied here formed a clade (marked in blue in Figures 1, 2) with eight other *Hoya* species, viz. *H. bella* Hook., *H. chinghungensis* (Tsiang & P.T. Li) M.G. Gilbert, P.T. Li & W.D. Stevens, *H. edeni* King ex Hook. f., *H. engleriana* Hosseus, *H. lanceolata* Wall. ex D.Don, *H. linearis* Wall. ex D. Don, *H. longicalyx* Wang Hui & E.F.Huang and *H. thailandica* Thaithong. This clade is in accordance with clade I circumscribed in Wanntorp et al. (2014) and it is strongly supported (BSs = 100%, PPs = 1.00) here in all analyses except in the ML analysis, based on the cpDNA dataset (BS = 51%). The *Hoya* species in this clade are mainly restricted to the subtropical foothills of the Himalayas and the Tibet-Sichuan Plateau (Wanntorp et al. 2014). Morphologically, species included in this clade usually have small leaves (no longer than 3 cm in length), flat-topped pseudumbels and non-persistent peduncles (own observation), except the two earliest divergent species *H. thailandica* and *H. edeni* that have large leaves and sub-hemispherical umbels. The new species is most closely related with. *H. chinghungensis*, *H. engleriana*, *H. lanceolata* and *H. longicalyx* (BSs = 84%, PPs = 1.00) in the analyses of both the nrDNA dataset (Figure 1a) and the combined dataset (Figure 2), but relationships amongst these species were not resolved or poorly supported.

**Figure 3.**
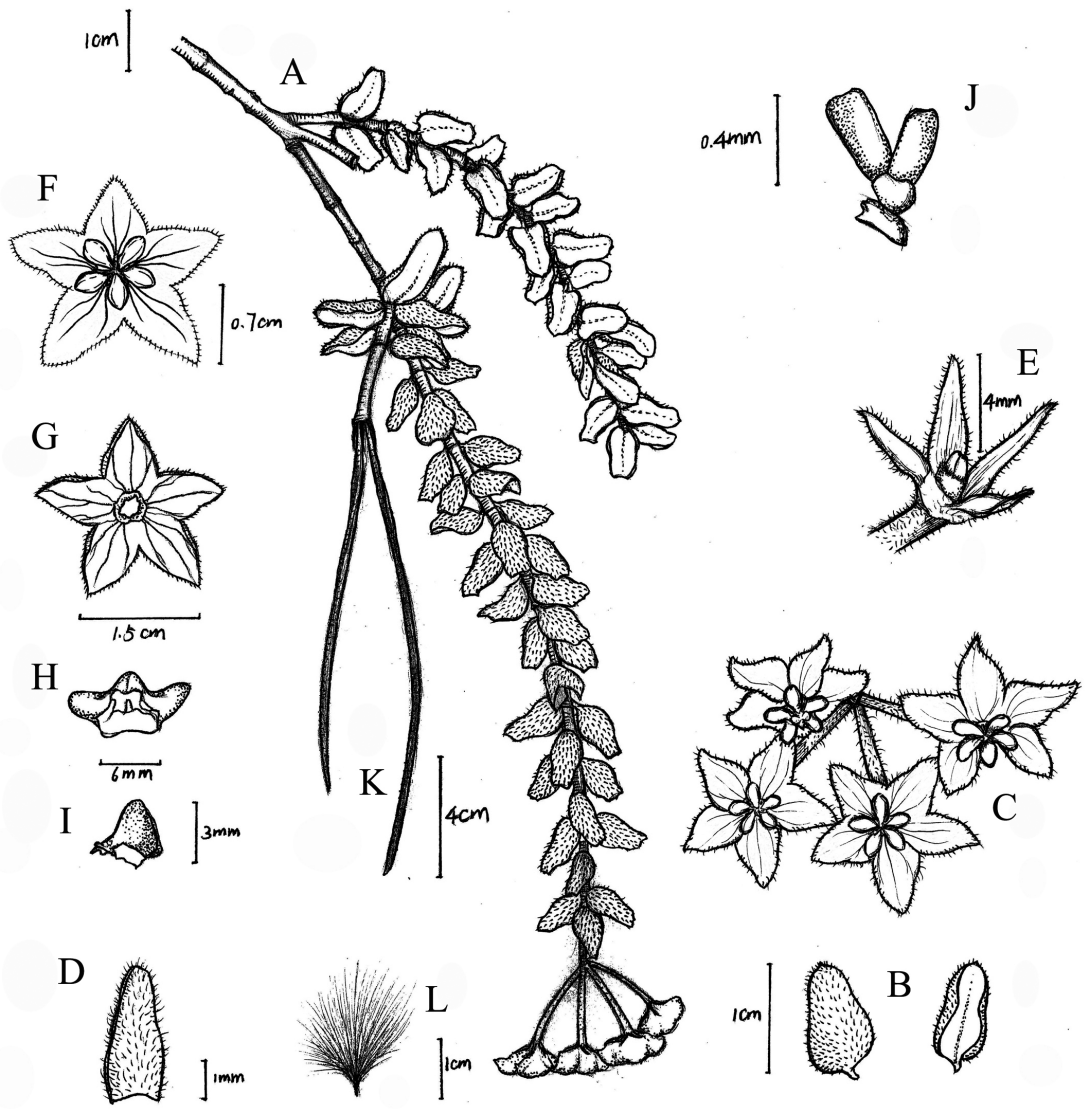
*Hoya pyrifolia* E.F. Huang **A** habit **B** leaf **C** inflorescence **D** bracteole **E** calyx lobes **F** corolla, adaxial side **G** corolla, abaxial side **H** corona **I** corona lobe **J** pollinarium **K** fruit **L** seed. Drawn by Y.J. Chen.

**Figure 4.**
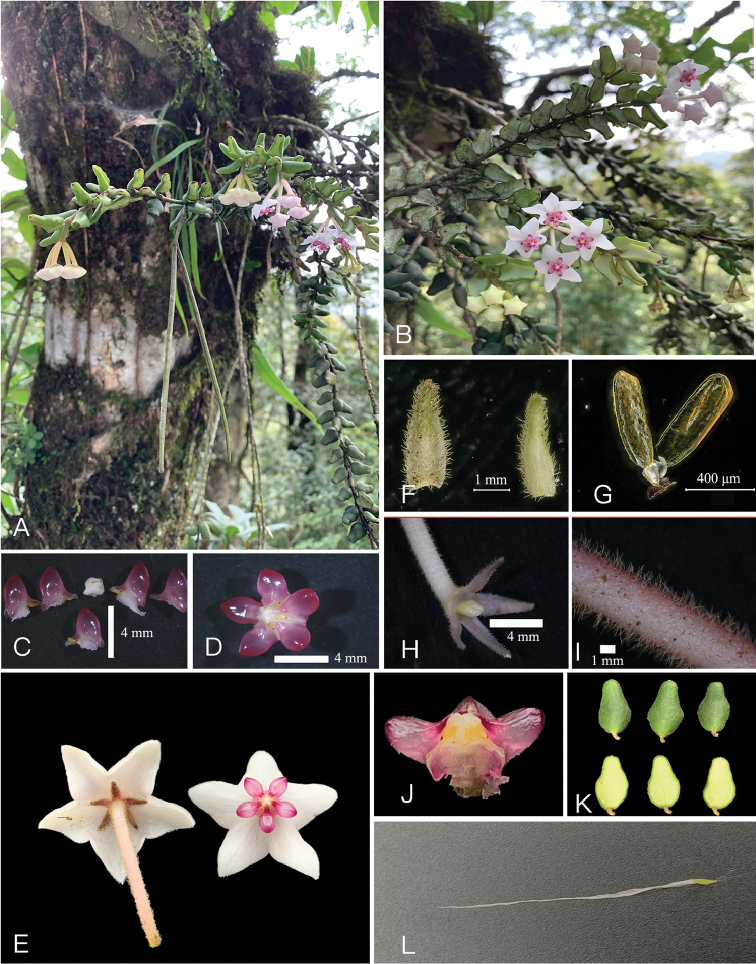
*Hoya pyrifolia* E.F. Huang **A** habit showing inflorescences and mature follicles **B** branch and inflorescence **C** corona lobes **D** corona top view **E** flower **F** bracteoles **G** pollinarium **H** pedicel and calyx lobes **I** part of Pedicel **J** corona side view **K** leaves **L** seed.

**Figure 5.**
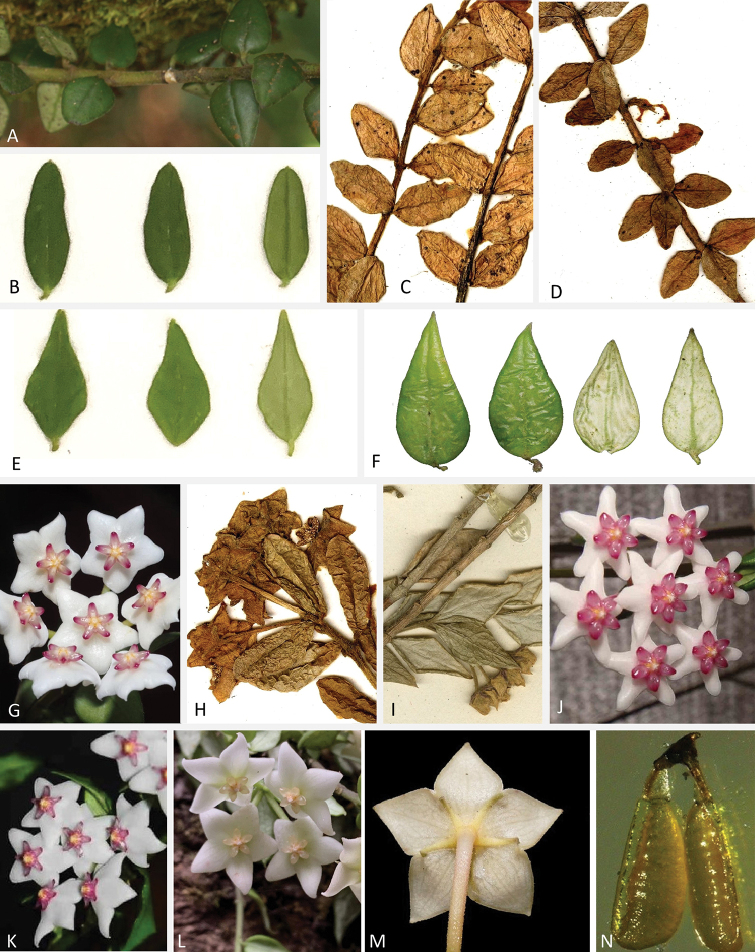
Leaves and inflorescences of *Hoya* species **A, G***H. chinghungensis* (Y. Tsiang & P.T. Li) M.G. Gilbert, P.T. Li & W.D. Stevens **B, J***H. engleriana* Hosseus; **C–D, H***H. dickasoniana* P.T. Li **E, K***H. lanceolata* Wall. ex D. Don; **F, L–N***H. longicalyx* W. Hui & E.F. Huang **I***H. kingdonwardii* P.T. Li.

Morphologically, the new species is similar to *H. engleriana* and *H. longicalyx*. However, it can be easily distinguished from the latter two species by a series of morphological traits (Figures 3, 4), such as its pyriform leaves that are 10–14 mm long (Figure 4A, B, K), with rounded or truncate leaf apex (Figure 4K) and mid-vein invisible adaxially and obscure abaxially (Figure 4K), the 4-flowered inflorescences (Figure 4A, B) with 8–10 mm long peduncles, the ca. 4 mm long calyx lobes (Figure 4H), the triangular corolla (Figure 4E), the rose-coloured corona (Figure 4C–E, J) and the oblong and ca. 0.6 mm long pollinia (Figure 4G). In contrast, *H. engleriana* is characterised by its narrowly-oblong leaves that are 20–25 mm long (Figure 5B) with usually acute or obtuse leaf apex (Figure 5B) and mid-vein evident on both surfaces (Figure 5B), the 5–7-flowered inflorescences (Figure 5J), the 1.5–2 mm long calyx lobes (Huang et al. 2020a) and the narrowly-oblong to oblong-triangular corolla (Figure 5J); *H. longicalyx* is characterised by its ovate-lanceolate leaves that are 15–20 mm long (Figure 5F) with acuminate leaf apex (Figure 5F) and mid-vein depressed adaxially and raised abaxially (Figure 5F), the ca. 5 mm long peduncles (Huang et al. 2020a), the 5–7 mm long calyx lobes (Huang et al. 2020a; Figure 5M), the whitish corona (Figure 5L) and the clavate pollinia narrowing towards the base (Figure 5N).

The new species is also similar to *H. dickasoniana* P.T.Li and *H. kingdonwardii* P.T.Li in morphology. The two latter species were described from Myanmar, but are not included in the phylogenetic analyses due to lack of DNA material. According to the protologues and holotypes of these two Burmese endemic species, the new species studied here can be distinguished from them by its opposite leaf arrangement, pyriform and slightly pubescent leaves (Figure 4A, B, K) with obtuse or rounded leaf base and rounded to truncate apex (Figure 4K), 4-flowered inflorescences (Figure 4A, B) with 8–10 mm long peduncles and pollinia that are ca. 0.6 mm long (Figure 4G). In contrast, *H. dickasoniana* is characterised by its leaf arrangement which is opposite or in whorls of 3–4 (Figure 5C, D), the leaves which are elliptic to ovate and glabrous on both surfaces (Figure 5C, D) with leaf base broadly cuneate to rounded and apex obtuse (Figure 5C, D), the inflorescence with up to 6 flowers (Figure 5H) and ca. 5 mm long peduncles (Li 1994; Figure 5H) and the ca. 1 mm long pollinia (Li 1994), while *H. kingdonwardii* is characterised by its leaves that are elliptic to slightly elliptic-lanceolate and glabrous on both surfaces (Li 1994; Figure 5I) with cuneate base and acuminate apex (Figure 5I) and mid-vein evident on both surfaces (Figure 5I) and the ca. 0.8 mm long pollinia (Li 1994).

Detailed information about the morphological comparison between the new species and its close relatives are given in Table 1.

**Table 1.** Morphological comparison between *Hoya pyrifolia* E.F.Huang, its closest relatives and morphologically-similar species.

Taxa	Leaf	Inflorescence	Corona	Calyx lobes	Pollinia
*H. chinghungensis*	Ovate to broadly ovate, 10–13 mm × 7–10 mm; pubescent on both surfaces when young; base rounded to truncate, apex rounded to obtuse or acuminate; midvein evident on both surfaces	4–5-flowered; peduncle ca. 8 mm long	Rose-colored	1.5–2 mm long	Unknown
*H. dickasoniana*	Elliptic or ovate, 9–19 mm × 5–10 mm; glabrous; base broadly cuneate to rounded, apex obtuse; midvein evident on both surfaces	3–6-flowered peduncle ca. 5 mm long	Unknown	2–2.5 mm long	Oblong; ca. 1 mm long;
*H. engleriana*	Narrowly oblong, 20–25 mm × ca. 5 mm; pubescent on both surfaces when young; base cuneate, apex usually obtuse with mucro; midvein evident abaxially	5–7-flowered; peduncle ca. 6 mm	Rose-colored	1.5–2 mm long	Unknown
*H. kingdonwardii*	Elliptic to slightly elliptic-lanceolate, 10–17 mm × 5–10 mm; glabrous; base cuneate and apex acuminate; midvein evident on both surfaces	3–4-flowered; peduncle 7–10 mm long	Unknown	ca. 3 mm long	Oblong; ca. 0.8 mm long
*H. lanceolata*	Lanceolate, ca. 25 mm × 15 mm; based cuneate to narrowly cuneate, apex acuminate; sparsely pubescent on both surfaces when young; midvein evident on both surfaces;	7–12-flowered; peduncle ca. 12 mm	Rose-colored	Unknown	Unknown
*H. longicalyx*	Ovate-lanceolate, 15–20 mm × ca. 10 mm; base rounded, apex acuminate; slightly pubescent; midvein depressed adaxially, raised abaxially;	3–4-flowered; peduncle ca. 5 mm long	Whitish	5–7 mm long	Clavate; 0.55–0.6 mm long, narrowing towards the base;
*H. pyrifolia*	Pyriform, 10–14 mm × 4–7 mm; slightly pubescent adaxially, glabrous abaxially; base obtuse or rounded, apex rounded or truncate; midvein absent adaxially, obscure abaxially	4-flowered; peduncle 8–10 mm long	Rose-colored	ca. 4 mm long	Oblong; ca. 0.6 mm long

### Taxonomic treatment

#### Hoya pyrifolia

Taxon classificationPlantaeGentianalesApocynaceae

E.F. Huang sp. nov.

7EF76E05-95FF-5CA1-B520-31D0430F40A0

urn:lsid:ipni.org:names:77215716-1

Figures 3 , 4

##### Diagnosis.

The species is morphologically most similar to *H. engleriana*, but differs by its leaves which are pyriform and 10–14 mm long (vs. narrowly oblong and 20–25 mm long), its 4-flowered inflorescences (vs. 5–7-flowered), its calyx lobes ca. 4 mm long (vs. 1.5–2 mm long) and the triangular corolla (vs. narrowly oblong to oblong-triangular).

##### Type.

CHINA. Yunnan Province, Yingjiang Hsien, Sudian Village, Mulonghe River, epiphytic on trunk in mid-montane evergreen forest, 25°9'38"N, 97°53'20"N, at an elevation of 1865 m, 13 August 2019, E.F. *Huang 1905009* (Holotype: PE!; isotypes: PE!).

##### Description.

Epiphytic shrubs. Stems up to 60 cm in length, 3–4 mm in diam., branching mainly near base, branches pubescent, internodes shorter than leaves. Leaves opposite, pyriform, 10–14 × 4–7 mm, fleshy, slightly pubescent and dark green adaxially, glabrous and greyish-green abaxially, base obtuse or rounded, apex rounded or truncate, margin entire and reflexed; mid-vein invisible adaxially, obscure abaxially, lateral veins invisible on both surfaces; petioles ca. 2 mm long. Inflorescences terminal pseudumbels, flat-topped, 4-flowered, pendent; peduncle shorter than pedicels, 8–10 mm long, light green; bracteoles 2 at each pedicel base, linear, 4 × 1 mm; pedicels 1.3–1.5 cm long, light pink to yellow-green, pubescent; calyx lobes pinkish, narrowly triangular to linear, 4 × 1 mm, margin entire; corolla white, flat to slightly incurved, 1.5–1.7 cm in diam., lobes triangular-ovate, ca. 7 mm wide, apex acute; corona rose-coloured, ca. 6 mm in diam., ca. 3 × 3 mm, scales 5, fleshy, translucent, ovate-triangular; pollinia oblong, ca. 0.6 × 0.2 mm, base and apex truncate, caudicula attached at the centre of the retinaculum. Ovaries 2, attached to each other below centre, free higher up, oblong, ca. 2 mm long, ca. 1 mm wide, yellowish-white, pubescent. Follicles linear, 10–12 cm long, pubescent. Seeds linear-oblong, ca. 2.0 × 0.2 mm, coma 2.8–3.0 cm.

##### Distribution and habitat.

The species is endemic to Gaoligong Mountain, distributed in Longling and Yingjiang Counties in Yunnan Province. It is an epiphyte on tree trunks in the mid-montane evergreen forests at an elevation from 1850 m to 2150 m.

##### Etymology.

*Hoya pyrifolia* is named for its pyriform leaf, which is a significant feature that can be used to distinguish the species from its close relatives.

##### Other specimen examined.

CHINA. Yunnan Province, Longling Hsien, Gaoligongshan National Forest Park, 4°50'3"N, 98°45'48"E, at an elevation of 2146 m, 26 August 2019, *E.F. Huang 201908260012* (IBSC).

## Supplementary Material

XML Treatment for Hoya pyrifolia

## Appendix 1

GenBank accession numbers for samples used in this study (—, missing data; *, newly-generated sequences). GenBank accession numbers are given for ETS, ITS, *matK*, *psbA-trnH* and *trnT-trnL*.

***Hoya affinis*** Hemsl., HE794222, DQ334481, HQ327536, HQ327615, HE794732; ***H. albiflora*** Zipp. ex Blume, HE794249, DQ334493, HQ327567, HQ327647, HE794761; ***H. anulata*** Schltr., HE794280, DQ334485, HQ327584, HQ327663, HE794794; ***H. archboldiana*** C.Norman, HE794258, HQ327513, HQ327566, HQ327646, HE794770; ***H. ariadna*** Decne., HE794223, DQ334502, HQ327535, HQ327614, HE794733; ***H. bella*** Hook., HE794215, HQ327518, HQ327581, HQ327660, HE794725; ***H. bilobata*** Schltr., HE794296, HE794381, HQ327599, HQ327678, HE794811; ***H. bordenii*** Schltr., HE794344, HQ327527, HQ327607, HQ327686, HE794862; ***H. campanulata*** Blume, HE794242, HE794387, MH598655, HE794484, HE794754; ***H. carnosa*** (L.f.) R.Br., HE794320, DQ334460, HQ327586, HQ327665, HE794837; ***H. chinghungensis*** (Y.Tsiang & P.T.Li) M.G.Gilbert, P.T.Li & W.D.Stevens, MN089474, MN107851, MT300095, MK361041, MT300101; ***H. ciliata*** Elmer ex C.M.Burton, HE794224, DQ334512, HQ327537, HQ327616, HE794734; ***H. cinnamomifolia*** Hook., HE794328, HQ327510, HQ327562, HQ327642, HE794845; ***H. citrina*** Ridl., HE794329, HQ327507, HQ327558, HQ327638, HE794846; ***H. cominsii*** Hemsl., HE794356, HQ327515, HQ327577, HQ327656, HE794873; ***H. cumingiana*** Decne., HE794275, HQ32752, HQ327594, HQ327673, HE794788; ***H. curtisii*** King & Gamble, HE794279, DQ334479, HQ327608, HQ327687, HE794792; ***H. dennisii*** P.I.Forst. & Liddle, HE794252, HQ327524, HQ327543, HQ327622, HE794764; ***H. dimorpha*** F.M.Bailey, HE794259, HQ327512, HQ327565, HQ327645, HE794771; ***H. diptera*** Seem., HE794285, HQ327519, HQ327583, HQ327662, HE794798; ***H. edeni*** King ex Hook.f., HE794216, DQ334476, HQ327582, HQ327661, HE794726; ***H. engleriana*** Hosseus, MT300093, MG649233, MT300096, —, MT300102; ***H. erythrostemma*** Kerr, HE794325, HQ327511, HQ327563, HQ327643, HE794842; ***H. finlaysonii*** Wight, HE794333, HQ327506, HQ327557, HQ327637, HE794850; ***H. heuschkeliana*** Kloppenb., HE794299, DQ334416, HQ327542, HQ327621, E794780; ***H. imbricata*** Decne., HE794314, DQ334480, HQ327605, HQ327684, HE794831; ***H. ischnopus*** Schltr., HE794260, HE794411, MH598707, HE794507, HE794772; ***H. kentiana*** C.M.Burton, HE794300, DQ334424, HQ327598, HQ327677, HE794815; ***H. kerrii*** Craib, HE794318, DQ334458, HQ327545, HQ327624, HE794835; ***H. lacunosa*** Blume, HE794310, DQ334499, HQ327601, HQ327680, HE794827; ***H. lanceolata*** Wall. ex D.Don, MT300094, —, MT300097, MT300099, MT300103; ***H. linearis*** Wall. ex D.Don, HE794218, HQ327517, MH598727, HQ327659, HE794728; ***H. lobbii*** Hook.f., HE794365, HE794420, MH598726, HE794516, HE794882; ***H. longicalyx*** Wang Hui & E.F.Huang, MN268319, MN116005, MT300098, MT300100, MT300104; ***H. macgillivrayi*** F.M.Bailey, HE794262, DQ334488, HQ327568, HQ327648, HE794774; ***H. meliflua*** Merr., HE794315, DQ334429, HQ327571, HQ327651, HE794832; ***H. merrillii*** Schltr., HE794349, HQ327505, HQ327555, HQ327635, HE794866; ***H. mitrata*** Kerr, HE794309, DQ334500, HQ327604, HQ327683, HE794826; ***H. multiflora*** Blume, HE794238, DQ334487, HQ327550, HQ327629, HE794750; ***H. naumannii*** Schltr., HE794265, HE794434, MH598742, HE794529, HE794777; ***H. odorata*** Schltr., HE794277, HQ327504, HQ327554, HQ327634, HE794790; ***H. onychoides*** P.I.Forst., Liddle & I.Liddle, HE794248, HE794439, MH598748, HE794533, HE794760; ***H. patella*** Schltr., HE794255, DQ334498, HQ327553, HQ327633, HE794767; ***H. polyneura*** Hook.f., HE794366, HQ327509, HQ327560, HQ327640, HE794883; ***H. praetorii*** Miq., HE794237, HQ327520, HQ327585, HQ327664, HE794749; ***H. puber*** Blume, HE794287, HQ327526, HQ327544, HQ327623, HE794800; ***H. pubicalyx*** Merr., HE794321, DQ334447, HQ327588, HQ327666, HE794838; ***H. pyrifolia*** E.F.Huang, MW066464*, MW035791*, MW066465*, MW066466*, MW066467*; ***H. retusa*** Dalzell, HE794364, DQ334457, MH598775, HQ327632, HE794881; ***H. rubida*** Schltr., HE794266, HE794453, MH598777, HE794545, HE794778; ***H. serpens*** Hook.f., HE794368DQ334482, HQ327575, HQ327654, HE794885; ***H. siariae*** Kloppenb., HE794355, HE794459, JX120795, HE799701, HE794870; ***H. sussuela*** Merr., HE794233, HE794464, MH598784, HE794559, HE794743; ***H. telosmoides*** Omlor, HE794240, DQ334486, HQ327592, HQ327671, HE794752; ***H. thailandica*** Thaithong, HE794219, HE794466, —, HE794561, HE794729; ***H. thomsonii*** Hook.f., HE794369, HQ327502, HQ327546, HQ327625, HE794886; ***H. vitellina*** Blume, HE794342, HQ327508, HQ327559, HQ327639, HE794859; ***H. waymaniae*** Kloppenb., HE794244, HQ327522, HQ327596, HQ327675, HE794756; ***Marsdenia laxiflora*** Donn.Sm., MK214583, LR794776, MH748870, —, LR794694.
